# Effectiveness of Various Light-Cure Biomaterials as an Intraorifice Barrier: An In Vitro Study

**DOI:** 10.7759/cureus.78587

**Published:** 2025-02-05

**Authors:** Surender Arumugam, Sebeena Mathew, Sankar Vishwanath, Santhiya Ravichandran, Karthick Kumaravadivel, Boopathi Thangavel

**Affiliations:** 1 Conservative Dentistry and Endodontics, KSR Institute of Dental Science and Research, Tiruchengode, IND

**Keywords:** bulk-fill composite, composite resins, coronal seal, dye extraction, intraorifice barrier, microleakage, push-out, rmgic, sealing ability, theracal

## Abstract

Objective

Proper seal achieved via placement of an effective intraorifice barrier curtails the occurrence of post-bleaching consequences and reinforces the cervical tooth structure. Hence, our present study aims to evaluate the effectiveness of three different light-cure biomaterials as an intraorifice barrier.

Methodology

Forty-eight sound human premolars were obtained, and root canal treatment procedures were accomplished according to the standard protocol. Gutta-percha was removed from the cementoenamel junction to a uniform depth of 3 mm, and the barrier materials were placed according to the respective groups (n=16 each): Group I: resin-modified glass ionomer cement (RMGIC) (Ionoseal, VOCO, Innovative Biotherapies, Inc., Ann Arbor, Michigan, United States), Group II: TheraCal LC (Bisco, Inc., Schaumburg, Illinois, United States), and Group III: bulk-fill composite (BFC) (Tetric N-Ceram, Ivoclar Vivadent, Inc., Schaan, Liechtenstein). Eight samples from each group were coated with nail varnish, submerged in 2% methylene blue for 24 hours, and evaluated for sealing ability using ultraviolet-visible (UV-Vis) spectrophotometry. The remaining eight samples from each group were subjected to push-out bond strength evaluation using the universal testing machine. Values were statistically analyzed using one-way ANOVA and Scheffe's t-test.

Result

TheraCal LC showed the highest mean value of sealing ability to other groups; however, there is an insignificant difference compared with BFC. Push-out bond strength values are increasing, with Group II being the lowest, followed by Group I and the BFC group demonstrating superior performance.

Conclusion

TheraCal LC exhibited the least microleakage among the experimental groups. Yet BFC stands out to be a reliable material of choice owing to its superior strength and fairly acceptable sealing ability.

## Introduction

The growing trends in aesthetic dentistry have driven patients to be solicitous about getting an ideal smile. Dyschromia of an anterior tooth is often associated with cosmetic issues of concern [1]. There are several methods for managing discolorations in vital and non-vital teeth. Walking bleach, a minimally invasive variant of internal tooth bleaching, is an affordable, safe, and effective treatment for discolored endodontically treated teeth [2]. A proper cervical barrier is imperative to eliminate any postoperative complications by preventing bleaching agents from penetrating the periapex and the external tooth surface [3].

In the literature, numerous materials have been studied and reported as protective intraorifice barriers. Glass ionomer cement (GIC), resin-modified glass ionomer cement (RMGIC), bulk-fill composite (BFC), and bioceramic materials like mineral trioxide aggregate (MTA) and Biodentine™ (Septodont, Saint-Maur-des-Fossés, France) are no exceptions [4].

TheraCal LC (Bisco, Inc., Schaumburg, Illinois, United States), a modified calcium silicate-based material indicated primarily for pulp capping and liner for restorative purposes, was combined with a resin component that allows for command setting of the material, offering several key benefits to the patient as well as the clinician. This injectable material has been studied as a pulp-capping agent and has proven to be effective [5]. The perforation-sealing ability of this bio-interactive material has been evaluated in previous studies; hence, our study intended to evaluate its role as an intraorifice barrier in comparison with other light-cure biomaterials like RMGIC and BFC. The aim of this in vitro study is to evaluate the sealing ability and push-out bond strength of the biomaterials as intraorifice barriers.

## Materials and methods

An in vitro study was designed and conducted at the KSR Institute of Dental Science and Research in Tiruchengode, India, after obtaining approval from the institute's Institutional Review Board (approval number: 14/KSRIDSR/IRB/2025). Sample size estimation was done using the G*Power software (Version 3.1, Heinrich-Heine-Universität Düsseldorf, Düsseldorf, Germany). With an effect size (f) of 0.467, α error of 5% (0.05), and actual power of 0.95, the total sample size was estimated to be 48.

Sample preparation

After obtaining ethical clearance, 48 sound human premolars extracted for periodontal and orthodontic reasons were collected. Samples were stored in saline until further use. The included samples were caries-free single-rooted premolars devoid of developmental defects, fractures, and cracks. Those with calcified canals and supernumerary and dilacerated roots were excluded from the study. Samples were wax-mounted during the procedure. Root canal configuration was confirmed with a preoperative radiograph, and access opening was performed using a round bur under high speed with water coolant. Working length was determined, and biomechanical preparation was carried out till the F2 size of the ProTaper Gold rotary files (Dentsply Sirona, Inc., Charlotte, North Carolina, United States). Between each instrument, 3% sodium hypochlorite (NaOCl) (Prime Dental Products, Inc., Thane, India) was used for irrigation. Final rinsing was done with 2 ml of 17% ethylenediaminetetraacetic acid (EDTA) (MD-Cleanser, Meta Biomed, Cheongju-si, Chungcheongbuk-do, Republic of Korea) and 5.25% NaOCl (Prime Dental Products, Inc.). After drying with ProTaper Gold paper points (Dentsply Sirona, Inc.), root canal samples were obturated with AH Plus resin-based sealer (Dentsply Sirona, Inc.) and F2-size gutta-percha (Dentsply Sirona, Inc.). Accessory cones were used to fill the spaces using the cold lateral compaction technique.

Using a Gates Glidden drill (Mani, Inc., Utsunomiya, Tochigi, Japan) size no. 5, coronal gutta-percha was removed to a depth of 3 mm from the cementoenamel junction, facilitating 1.3 mm at its widest point. This corresponds to the standardized thickness of barrier material placed in all the samples. The prepared samples were allocated into respective groups.

Barrier placement

Tooth samples were divided according to three intraorifice barrier materials into the following groups (n=16): Group I, RMGIC (Ionoseal, VOCO, Innovative Biotherapies, Inc., Ann Arbor, Michigan, United States), was injected into the prepared space without voids and light cured for 20 seconds. Group II, TheraCal LC (Bisco, Inc.), was injected into the prepared space and light cured for 20 seconds. In Group III, BFC (Tetric N-Ceram, Ivoclar Vivadent, Inc., Schaan, Liechtenstein), the prepared space was etched for 20 seconds (N-Etch, Ivoclar Vivadent, Inc.), adhesive (Te‑Econom Bond, Ivoclar Vivadent, Inc.) was applied in two coats and light cured for 15 seconds, and a 3 mm bulk composite material (Tetric N-Ceram, Ivoclar Vivadent, Inc.) was placed and cured for 60 seconds. All the abovementioned procedures were performed by a single trained operator to avoid interoperator variability. Half of the samples were subjected to the dye extraction method followed by ultraviolet-visible (UV-Vis) spectrophotometry, and the other half were used to assess the push-out bond strength.

Dye extraction method

Eight specimens from each group were kept at 100% humidity in an incubator at 37°C for 24 hours. External root surfaces of all the specimens were uniformly coated with multiple layers of nail varnish from the level of the cementoenamel junction to the root apex. The inner surface of the pulp chamber is exposed to 2% methylene blue dye (Sigma-Aldrich, Inc., St. Louis, Missouri, United States), for 24 hours. Then the samples were cleansed in running tap water for 30 minutes, and all the residual dye materials were removed and kept in vials containing 65% nitric acid. After three days, the vials were centrifuged for five minutes at 14,000 rpm. To assess the microleakage, 2 ml of the supernatant was collected and examined at a wavelength of 550 nm in a UV-Vis spectrophotometer (UV-1800, Shimadzu Analytical Pvt. Ltd., Mumbai, India). Readings were noted as absorbance units.

Push-out bond strength testing

The remaining eight samples of each group were mounted in a resin block and transversely sectioned using a cutting saw at high speed under water coolant. The dimensions of the slices corresponded to the intraorifice barrier and were of uniform 2 mm thickness, corresponding to the intraorifice barrier.

The specimens were positioned in the jigs of a universal testing machine (UTM) (Tecsol TSI-BDS-2kN, Chennai, India) and subjected to a static vertical load of 50 kg. A stainless-steel plunger of 0.7 mm diameter was driven at a crosshead speed of 0.5 mm/min until the material dislodged. The plunger contacted only the material and had a 0.2 mm clearance from the dentin. The UTM was interfaced with the computer software, and the bond strength analysis was performed. The highest load-to-dislodgment value was recorded and tabulated in megapascals (MPa). 

Statistical analysis

Statistical analysis was performed using IBM SPSS Statistics for Windows, Version 25.0 (Released 2017; IBM Corp., Armonk, New York, United States). The normality of data distribution was assessed using the Shapiro-Wilk test. All values indicated that the data followed a normal distribution; therefore, parametric methods were used for comparisons. Mean values between groups were compared using one-way ANOVA, followed by post hoc analysis with Scheffe's t-test. A p-value of less than 0.05 (p<0.05) was considered statistically significant.

## Results

**Figure 1:**
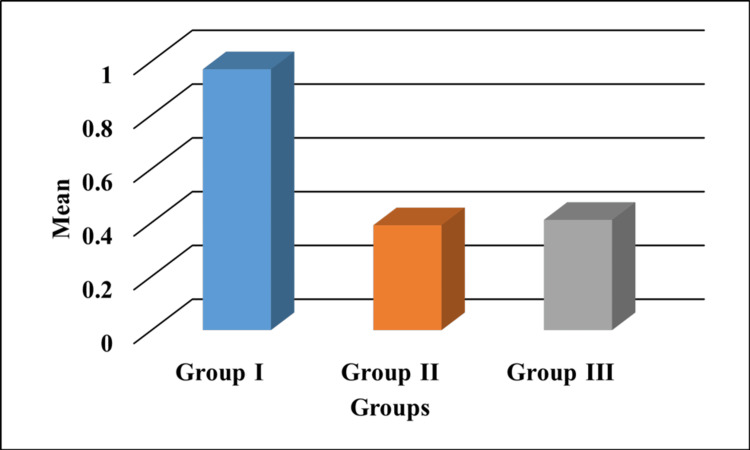
Sealing ability values of all the groups represented in AU. Group I: RMGIC; Group II: TheraCal LC; Group III: BFC The y-axis represents the mean absorbance value (AU). RMGIC: resin-modified glass ionomer; BFC: bulk-fill composite; AU: absorbance unit

Figure 1 represents the sealing ability values obtained by measuring the amount of dye extracted from the test samples. Absorbance units were in increasing order, RMGIC<BFC<TheraCal LC.

**Figure 2:**
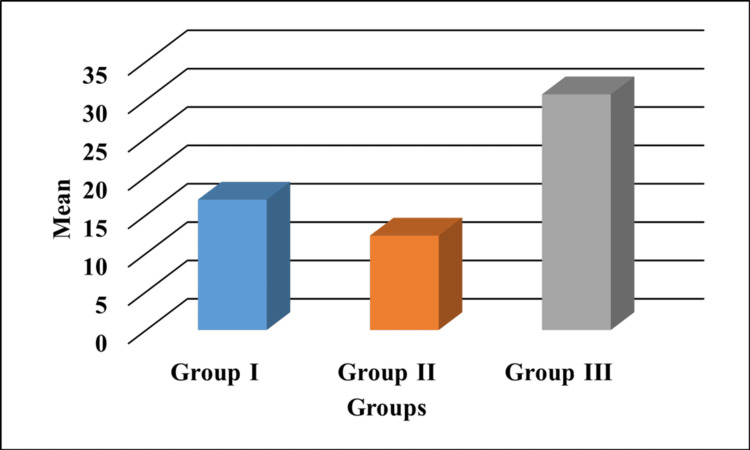
Graphical representation of push-out bond strength values expressed in MPa. Group I: RMGIC; Group II: TheraCal LC; Group III: BFC The y-axis indicates the mean bond strength values represented in MPa. RMGIC: resin-modified glass ionomer; BFC: bulk-fill composite; MPa: megapascals

Figure 2 depicts the load-to-displacement values of the test materials. The push-out bond strength was in the increasing order of TheraCal LC<RMGIC<BFC.

The post hoc values of push-out bond strength (Table 1) reveal a highly significant difference for Group III compared with Groups I and II, indicating the superior performance of BFC, whereas the post hoc values of the sealing ability of BFC (Table 2) show no statistical significance with Group II.

**Table 1:** One-way ANOVA followed by Scheffe's test (post hoc) for push-out bond strength values. Group I: RMGIC; Group II: TheraCal LC; Group III: BFC * denotes high statistical significance (p<0.001). RMGIC: resin-modified glass ionomer; BFC: bulk-fill composite; SD: standard deviation

Groups	Push-out (mean±SD)	F value	Group comparison	P-value
Group I	16.99±5.71	18.40	G-I with G-II	0.351
			G-I with G-III	0.001*
Group II	12.31±4.62		G-II with G-I	0.351
			G-II with G-III	0.0001*
Group III	30.72±8.08		G-III with G-I	0.001*
			G-III with G-II	0.0001*

**Table 2:** One-way ANOVA followed by Scheffe's test (post hoc) for sealing ability values. Group I: RMGIC; Group II: TheraCal LC; Group III: BFC * denotes high statistical significance (p<0.001). RMGIC: resin-modified glass ionomer; BFC: bulk-fill composite; SD: standard deviation

Groups	Sealing ability (mean±SD)	F value	Group comparison	P-value
Group I	0.97±0.32	19.97	G-I with G-II	0.0001*
			G-I with G-III	0.0001*
Group II	0.39±0.07		G-II with G-I	0.0001*
			G-II with G-III	0.980
Group III	0.41±0.12		G-III with G-I	0.0001*
			G-III with G-II	0.980

## Discussion

Proper sealing of the root canal space after disinfection becomes a mandatory requisite for a predictable treatment outcome. Most investigations have been targeted toward developing better techniques and materials to reinforce the apical seal. However, the long-term failures were mostly attributed to the inadequate coronal seal [6].

Despite its role in serving as a protective barrier in non-vital bleaching, providing a double-sealed coronal restoration over the gutta-percha will be a suitable strategy to reinforce the core build-up by preventing coronal leakage [7]. These double-sealed coronal restorations increase fracture resistance by reinforcing the cervical tooth structure and also reduce the chance of reinfection by sealing the root canals coronally [6].

A few important ideal requisites that an intraorifice barrier must fulfill are its easy handling characteristics, bonding ability to the tooth structure, good strength, and sealing ability [7]. Due to its ability to expand during the hydration reaction, conventional bioceramic materials like MTA can seal the interface effectively by chemical bond formation with the tooth structure [8]. However, one of the major drawbacks associated with the use of MTA is the extended setting time which makes it unsuitable as an intraorifice barrier material [9]. Hence, a command-setting material, TheraCal LC, a bioactive hybrid cement, that contains a light-curing resin component for enhanced setting is used as a barrier in this present study.

This bioceramic material has proven its role as an effective direct and indirect pulp capping material owing to its hydroxyapatite-forming ability contributing to its exquisite biological seal [5]. A scanning electron microscopic analysis by Nagmode et al. assessed the sealing abilities of MTA, Biodentine™, and a light-cure MTA-based material for furcal perforation repair and concluded its good sealing ability to dentin when compared to conventionally used Biodentine™ and MTA [10].

The most prevalent method for evaluating sealing ability is the dye penetration method; however, it is a qualitative method of assessment and has its limitations. Therefore, the dye extraction technique was applied in the current study. It recovers all the leaked dye by dissolving it in acid, thus eliminating the need to section the root. However, there are a few limitations in dye extraction such as variations in dye elution and the trapped dye particles that could affect this method's accuracy. Methylene blue dye was utilized to assess the sealing ability. Its characteristics such as its high degree of staining, ease of handling, and affordability highlight its use in the current study. Spectrophotometric analysis performed more accurately compared to the dye leakage methods [11].

There are conflicting opinions on the thickness of barrier materials; a recent systematic review by de Araújo et al. in 2022 confirmed that significantly improved results were obtained with an intraorifice barrier measuring 3 mm deep [8]. Therefore, the present study evaluated a barrier placement of a standard thickness of 3 mm.

From the results obtained, the BFC group exhibited superior push-out bond strength, which might be directly attributed to the bond strength of the adhesives and the high filler loading of the nanocomposite used in the present study [12]. No discernible differences in terms of the sealing ability were observed between BFC and TheraCal LC. This might be attributed to the presence of Ivocerin, a potent photoinitiator present in the nanofilled composite, which enhances the depth of cure to a greater extent. Its role as a polymerization modulator along with modified high-molecular-weight urethane dimethacrylate helps in reducing polymerization shrinkage and stress, thereby providing a better seal [13,14].

RMGIC possesses a modulus of elasticity presumably close to dentine, providing an even stress distribution in the cervical portion of the tooth before transmitting it to the root [15]. However, the major disadvantage of the resin component of RMGIC is polymerization shrinkage during the setting process. However, there exists a slight expansion of the glass ionomer matrix by absorbing water from dentinal tubules. Besides this inherent compensatory mechanism, a higher degree of microleakage was observed in the RMGIC group in the current study, and this is in coherence with the systematic review by de Araújo et al. [8]. Although there exists a compensatory expansion to overcome shrinkage, the clinical relevance of these dimensional changes remains questionable. 

Recent studies proposed various dentin treatments to enhance the compromised bond strength with TheraCal LC [16]. Conditioning of dentin or the addition of an acidic monomer improves the bonding without jeopardizing the mechanical properties of this material as an intraorifice barrier [17]. Though the bioactivity of the bioceramic material remains questionable, they may form a primary monoblock and exist as a single unit with the remaining tooth structure. This might strengthen the cervical tooth structure of endodontically treated teeth [16,18].

Clinical implications

Providing an effective barrier forms a primary step in determining the success of non-vital bleaching. This study highlights the use of light-cure biomaterials as an intraorifice barrier in providing an effective seal and also highlights its easy handling properties in clinical scenarios. 

Limitations

The effectiveness of other composite materials with varied filler contents as a barrier has to be evaluated since the current study employs only a nanofilled composite material. Long-term stress testing of these materials and the effects of thermocycling of these barriers need to be tested. Need for validation of the results of this study in clinical settings is necessary.

## Conclusions

Within the limitations of this study, it is concluded that TheraCal LC, a biocompatible bioceramic material, offered a good sealing ability and a comparatively lesser push-out bond strength than RMGIC and composite resin. In a clinical setting, a command set barrier material is the preferred choice due to its suitable working time. Despite not being a material that is frequently used as an intraorifice barrier, BFC is the dependable material of choice due to its superior strength and reasonably good sealing ability.
